# Additional Value of Non-contrast Chest CT in the Prediction of Adverse Cardiovascular Events in Patients With Novel Coronavirus Disease 2019 (COVID-19)

**DOI:** 10.3389/fcvm.2021.738044

**Published:** 2021-10-15

**Authors:** Shuang Li, Xiaojun Wang, Hongyao Hu, Jing Xu, Jian He, Wenjing Yang, Bin He, Yanmei Liu, Huidan Yu, Quan Zhou, Haijun Zhang, Tingting Liu, Ke Hu, Yang Zhao, Zhixin Huang, Hengcheng Zhu, Bicheng Zhang, Shihua Zhao, Arlene Sirajuddin, Andrew E. Arai, Jun Chen, Xiaoyang Zhou, Minjie Lu

**Affiliations:** ^1^Department of Magnetic Resonance Imaging, State Key Laboratory of Cardiovascular Disease, Fuwai Hospital, National Center for Cardiovascular Diseases, Chinese Academy of Medical Sciences and Peking Union Medical College, Beijing, China; ^2^Department of Internal Medicine, Maternal and Child Hospital of Hubei Province, Tongji Medical College, Huazhong University of Science and Technology, Wuhan, China; ^3^Department of Radiology, Renmin Hospital of Wuhan University, Wuhan, China; ^4^Department of Pediatrics, Renmin Hospital of Wuhan University, Wuhan, China; ^5^Pujiang Branch of the First Affiliated Hospital, School of Medicine, Cardiovascular Center of Middle Zhejiang, Zhejiang University, Jinhua, China; ^6^School of Health Science, Wuhan University, Wuhan, China; ^7^Department of Clinical Laboratory, Renmin Hospital of Wuhan University, Wuhan, China; ^8^Department of Cardiology, Renmin Hospital of Wuhan University, Wuhan, China; ^9^Department of Respiratory and Critical Care Medicine, Renmin Hospital of Wuhan University, Wuhan, China; ^10^Department of Gynecology, Renmin Hospital of Wuhan University, Wuhan, China; ^11^Department of Urology Surgery, Renmin Hospital of Wuhan University, Wuhan, China; ^12^Cancer Center, Renmin Hospital of Wuhan University, Wuhan, China; ^13^Department of Health and Human Services, National Heart, Lung, and Blood Institute, National Institutes of Health, Bethesda, MD, United States; ^14^Key Laboratory of Cardiovascular Imaging (Cultivation), Chinese Academy of Medical Sciences, Beijing, China

**Keywords:** non-contrast chest CT, novel coronavirus disease 2019 (COVID-19), risk factors, cardiac injury, adverse cardiovascular events

## Abstract

**Background:** Coronavirus disease 2019 (COVID-19) has outbroken in China and subsequently spread worldwide since the end of 2019. Chest computed tomography (CT) plays an important role in the diagnosis of lung diseases, but its value in the diagnosis of cardiac injury remains unknown.

**Methods:** We enrolled 241 consecutive hospitalized patients (aged 61 ± 16 years, 115 males) with laboratory-confirmed COVID-19 at Renmin Hospital of Wuhan University from January 11 to March 2, 2020. They were divided into two groups according to whether major adverse cardiovascular events (MACEs) occurred during the follow-up. The anteroposterior diameter of the left atrium (LAD), the length of the left ventricle (LV), and cardiothoracic ratio (CTR) were measured. The values of myocardial CT were also recorded.

**Results:** Of 241 patients, 115 patients (47.7%) had adverse cardiovascular events. Compared with no MACEs, patients with MACEs were more likely to have bilateral lesions (95.7% vs. 86.5%, *p* = 0.01). In multivariable analysis, bronchial wall thickening would increase the odds of MACEs by 13.42 (*p* = 0.01). LAD + LV and CTR was the best predictor for MACEs (area under the curve = 0.88, *p* < 0.001) with a sensitivity of 82.6% and a specificity of 80.2%. Plasma high-sensitivity troponin I levels in patients with cardiac injury showed a moderate negative correlation with minimum CT value (*R*^2^ = −0.636, *p* < 0.001).

**Conclusions:** Non-contrast chest CT can be a useful modality for detection cardiac injury and provide additional value to predict MACEs in COVID-19 patients.

## Introduction

Coronavirus disease 2019 (COVID-19), a newly discovered infectious disease caused by severe acute respiratory syndrome coronavirus 2 (SARS-CoV-2), has spread rapidly around the world. The number of cases reported globally now exceeds 175 million (1). The common clinical manifestations are fever, cough, myalgia, or fatigue; some even have sputum production, headache, hemoptysis, and diarrhea (2). With the increase of confirmed cases and the deepening of research, it is found that the most common chronic comorbidities are cardiovascular diseases (such as hypertension, coronary artery disease), which had a high risk of death. Acute myocardial injury occurred in 12% of patients, second only to acute respiratory distress syndrome (29%) and RNAemia (15%) (2–4). Therefore, more and more attention has been paid to cardiovascular adverse events caused by viral infection. A meta-analysis shows that the values of cardiac troponin I (cTnI) were found to be significantly increased in COVID-19 patients with severe disease than in those without [standardized mean difference (SMD), 25.6 ng/L; 95% confidence interval (CI), 6.8–44.5 ng/L] (5). Shi et al. reported that patients with myocardial injury had a substantially higher in-hospital mortality rate (51.2%) compared with those without (4.5%), and greater degrees of TnI elevation were associated with higher mortality rates (6).

However, most of the current studies focus on the clinical characteristics and prognosis of patients with acute myocardial injury (diagnosed by elevation of troponin TnT/TnI levels), and there are few studies on the imaging findings of adverse cardiovascular events in patients with COVID-19. The majority of patients have undergone chest plain computed tomography (CT), but because of the limited tolerance of examination or economic factors, contrast-enhanced CT was rarely performed. In view of previous findings that a considerable number of patients have cardiovascular complications, which have a great impact on the prognosis (6–8), this study intends to detect the morphology and tissue characteristics of the heart by non–contrast-enhanced chest CT. Myocardial injury may cause myocardial edema. CT is sensitive to edema, which makes it possible for us to find myocardial damage. We hypothesized that besides evaluating lung lesions, a chest plain CT scan can provide additional information of cardiac injury and thereby predict the occurrence of adverse cardiovascular events in patients with COVID-19.

## Materials and Methods

### Study Design and Participants

We retrospectively enrolled 241 patients with laboratory-confirmed COVID-19 who were admitted to Renmin Hospital of Wuhan University from January 11 to March 2, 2020. COVID-19 was diagnosed with a positive result by real-time reverse transcriptase–polymerase chain reaction (RT-PCR) from clinical samples. Exclusion criteria were patients whose CT images were difficult to analyze because of poor quality. The study was approved by the Research Ethics Committee of the Renmin Hospital of Wuhan University, Wuhan, China (approval no. WDRY2020-K038). Written informed consent was waived by the ethics commission as it is a retrospective study. The procedures are in accordance with the Helsinki Declaration and the ethical standards of the human experiment responsibility committee.

The sample size was estimated based on the primary difference between the diameter of the left atrium (LAD) + left ventricle (LV) measurements. We assumed the mean and SD of the major adverse cardiovascular event (MACE) group were 12.1 and 1.5, respectively. The mean and SD of the control group were 11.4 and 1.3, respectively. The probability of type I error was 0.05 (with both sides), and the probability of type II error was 0.20. We calculated the necessary sample size for this research to be 126.

### Data Collection

Patient data including demographics, clinical characteristics, laboratory examinations, imaging findings, comorbidities, complications, treatment measures, and outcomes were collected from electronic medical records by two investigators (Xiaoyang Zhou and Tingting Liu). Chest CT was done for all the patients and reviewed by 2 radiologists independently (Shuang Li and Minjie Lu). The severity of COVID-19 was defined according to the Chinese management guideline for COVID-19 (version 7) (9). A patient was classified as “mild” if he had mild symptoms, with or without pneumonia on imaging. Patients who met any of the following criteria will be classified as “severe”: (1) shortness of breath, respiratory rate ≥30 times/min; (2) Sao_2_ ≤93% (at rest); (3) Pao_2_/Fio_2_ ≤300 mm Hg (1 mm Hg = 0.133 kPa). Patients who met any of the following criteria will be classified as “critical”: (1) respiratory failure that needs mechanical ventilation; (2) shock; (3) combined with other organ failure. Clinical outcomes were monitored until April 10, 2020. The endpoint was COVID-19–associated death. The discharge criteria included normal body temperature for more than 3 days, obvious improvement in respiratory symptoms and chest imaging, and at least two negative results for RT-PCR for COVID-19 (at least 24 h between sampling) (9). According to whether adverse cardiovascular events occurred during the follow-up, they were divided into two groups. The MACEs were heart failure, ischemic cardiovascular events, and malignant arrhythmia. Ischemic cardiovascular events include acute myocardial infarction and recurrent angina. Malignant arrhythmia was defined as rapid ventricular tachycardia lasting more than 30 s inducing hemodynamic instability and/or ventricular fibrillation. Cardiac injury was defined as blood levels of cardiac biomarkers [high-sensitivity TnI (hs-TnI)] above the 99th-percentile upper reference limit (0.04 ng/mL), regardless of new abnormalities in electrocardiography and echocardiography (10).

### CT Protocols and Image Analysis

Non-contrast chest CT scan was obtained with the patient in the supine position, and scanning was performed at end inspiration. To minimize motion artifacts, patients were instructed on breath-holding. CT examinations were done using Optima CT 680 (GE Healthcare, Bright Speed). The scans were acquired and reconstructed as axial images using the following parameters: 5.0-mm section thickness, 5.0-mm interval, 120 kVp, and adaptive tube current.

**Figure 1:**
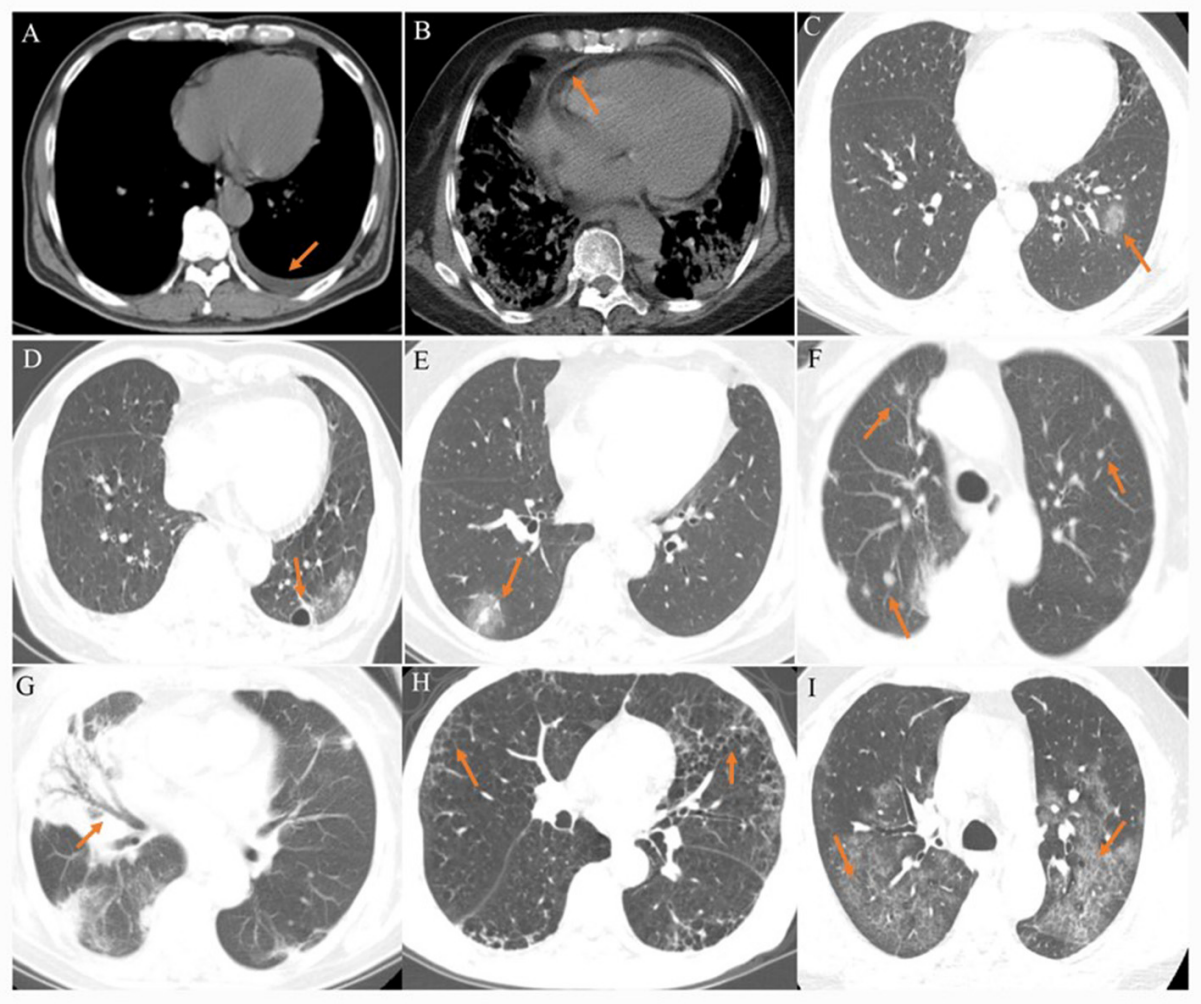
**(A)** A 40–45-year-old patient presenting fever for 5 days. CT scan shows left pleural effusion. **(B)** A 70–80-year-old patient presenting fever and shortness of breath for 10 days. CT scan shows pericardial effusion. **(C)** A 20–40-year-old patient presenting fever for 3 days. CT scan shows a ground-glass opacity in the left lower lobe. **(D)** A 40–45-year-old patient presenting fever with headache for 4 days. CT scan shows an air bubble sign in the left lower lobe. **(E)** A 30–50-year-old patient confirmed presenting fever for 4 days. CT scan shows a halo sign in the right lower lobe. **(F)** A 75–80-year-old patient presenting fever with cough for 9 days. CT scan shows bilateral multiple nodules. **(G)** A 40–45-year-old patient presenting fever for 8 days. CT scan shows air bronchogram in the middle lobe of right lung. **(H)** A 40–45-year-old patient presenting fever with cough for 5 days. CT scan shows bilateral reticular pattern. **(I)** A 40–45-year-old patient presenting fever with cough for 8 days. CT scan shows bilateral reticular pattern superimposed on the background of GGO, resembling the sign of crazy paving stones.

The CT images were assessed for the following features or patterns: (1) ground-glass opacity (GGO): hazy areas with slightly increased density in lungs without obscuration of bronchial and vascular margins (11); (2) consolidation: an increase in pulmonary parenchymal density with obscuration of bronchial and vascular margins, because alveolar air is replaced by pathological fluids, cells, or tissues (11); (3) reticular pattern: a collection of innumerable small linear opacities on CT images due to thickened pulmonary interstitial structures such as interlobular septa and intralobular lines (11, 12); (4) crazy paving pattern: thickened pulmonary interstitial structures with superimposition on a GGO background, resembling irregular paving stones (12); (5) air bronchogram: a pattern of air-filled bronchi on a background of opaque airless lung (11); (6) airway changes: include bronchiectasis and bronchial wall thickening; (7) pleural changes: including pleural thickening and pleural effusion; (8) fibrosis; (9) air bubble sign: a small air-containing space in the lung (12); (10) nodules: a rounded or irregular opacity with well- or poorly defined edges, measuring less than 3 cm in diameter (11); (11) halo sign: nodules or masses surrounded by ground glass (11); (12) lymphadenopathy; (13) pericardial effusion (Figure 1).

**Figure 2:**
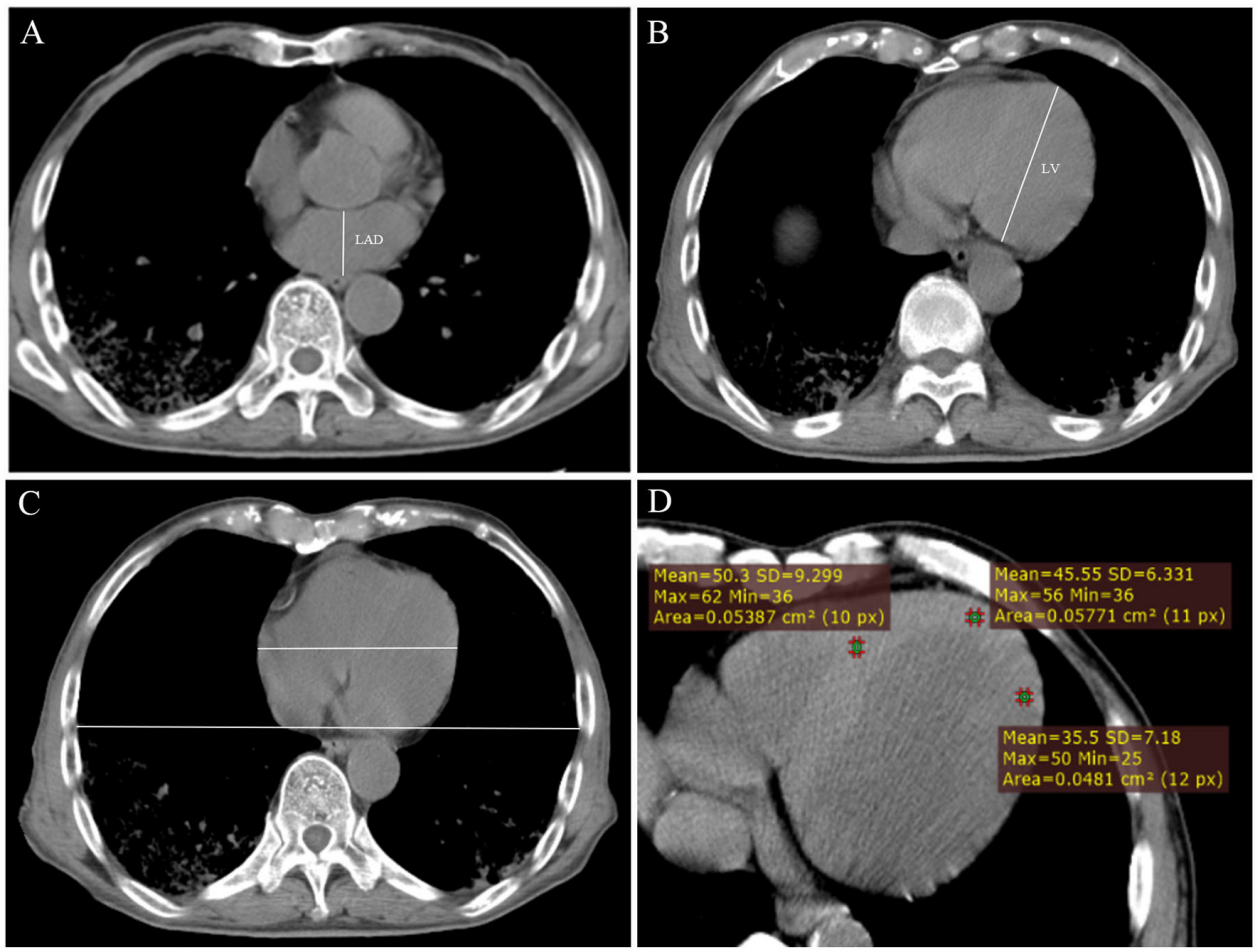
**(A)** Measurement of the anteroposterior diameter of the left atrium (LAD). **(B)** Measurement of the length of the left ventricle (LV). **(C)** Measurement of cardiothoracic ratio. **(D)** Measurement of CT value of myocardium at axial image.

The anteroposterior LAD and the length of the LV were measured. The sum of the two values was used as an index to evaluate the size of the heart. The cardiothoracic ratio (CTR) was also calculated. The slice in which the heart is the largest was selected; the maximum transverse diameter of the heart and the largest transverse diameter of the chest were measured, and the ratio of the two was calculated (Figure 2). We also measured the CT attenuation value of the myocardium: the median axial slice first (level 2) was select, and then the slices 4–10 mm above the median axial slice (level 1) and 4–10 mm below the median axial slice (level 3) were selected. Three regions of interest (ROIs) were drawn in these slices: the middle of the interventricular septum, the anterior wall of LV and the lateral wall of LV. The ROI should be round, with an area of 0.05 cm^2^. The mean, maximum, and minimum values of the myocardium were obtained (Figure 2).

### Statistical Analysis

Continuous variables were expressed as mean [standard deviation (SD)] or median [interquartile range (IQR)], and categorical variables were expressed as proportions. Student *t*-test, Mann–Whitney *U* test, and χ^2^ test or Fisher exact test were used to compare differences between patients with MACEs and patients without MACEs where appropriate. For survival analysis, Kaplan–Meier survival curves were compared between patients with and without MACEs using log-rank statistics. Survival values are expressed together with their 95% CIs defined as survival ±1.96 * SE. Univariable and multivariable logistic regression models were used to explore the risk factors associated with MACEs. Receiver operating characteristic curves were used to find the best image parameter prediction model to predict the MACEs during hospitalization. The cutoff value was identified using the Youden index, which is the maximum of (sensitivity + specificity – 1). The Spearman rank correlation coefficient was used for linear correlation analysis. Statistical analysis was performed using SPSS 23.0 software. Results were considered significant if *p* < 0.05.

## Results

### Demographic and Clinical Characteristics on Admission

We finally included 241 patients [aged 61 ± 16 years (29–98 years), 115 males] with laboratory-confirmed COVID-19, 115 (47.7%) of whom had MACEs, and 126 (52.3%) of whom did not. Most of the patients are in general [108 (44.8%)] and severe [109 (45.2%)] stage; only 24 (10.0%) cases were in critical stage. There were more severe and critical patients in MACE group than those without MACE group (52.2% vs. 38.9%, 16.5% vs. 4.0%, *p* < 0.001). The most common symptoms at admission were fever [186 (77.2%)], cough [133 (55.2%)], and fatigue [92 (38.2%)]. Shortness of breath was more common in patients with MACEs (33% vs. 21.4%, *p* = 0.04). The most common comorbidities were hypertension [96 (39.8%)], diabetes [41 (17.0%)], cardiovascular disease [39 (16.2%)], and cerebrovascular disease [13 (5.4%)], but only the differences in cardiovascular disease and cerebrovascular disease were statistically significant (21.7% vs. 11.1%, *p* = 0.049; 8.7% vs. 2.4%, *p* = 0.03). In terms of laboratory findings, patients with MACEs showed lower median lymphocyte counts [median (IQR), 0.9 (0.6–1.2) ×10^9^ cells/L vs. 1.4 (1.1–1.8) ×10^9^ cells/L, *p* < 0.001], platelets [median (IQR),198 (140–258) ×10^9^ cells/L vs. 225 (182–275) ×10^9^ cells/L, *p* = 0.01], hemoglobin [median (IQR),110 (95–125) g/dL vs. 117 (106–132) g/dL, *p* = 0.006], ALB [median (IQR),36.4 (32.8–39.5) g/dL vs. 38.9 (36.7–41.2) g/dL, *p* = 0.001), and higher level of C-reactive protein [median (IQR), 21.4 (5.1–67.5) mg/mL vs. 5.0 (5.0–24.2) mg/mL, *p* < 0.001] and d-Dimer [median (IQR), 6.4 (1.5–20.9) μg/mL vs. 0.96 (0.43–4.29) μg/mL, *p* < 0.001]. The cardiac markers in MACE group were higher than those without MACE group, and the difference was statistically significant, including CK-MB [median (IQR), 1.4 (0.8–3.4) ng/mL, *p* = 0.005], myoglobin [median (IQR), 49.1 (29.9–171.8) μg/L vs. 33.8 (21.7–53.2) μg/L, *p* < 0.001], hs-TnI [median (IQR), 0.027 (<0.006–0.183) ng/mL vs. 0.006 (<0.0060–0.0065) ng/mL, *p* = 0.021], N-terminal pro–B-type natriuretic peptide [median (IQR), 1910 (1055–5270) μg/mL vs. 216.4 (79.1–365.4] μg/mL, *p* < 0.001]. Furthermore, compared with patients without MACEs, more respiratory support treatment was applied in patients with MACEs. The detailed demographic data and clinical characteristics are shown in Table 1.

**Table 1:** Demographic, clinical, and laboratory findings of patients with COVID-19 on admission.

	**All (*n* = 241)**	**MACEs (*n* = 115)**	**No MACEs (*n* = 126)**	***p***
Age	61 (29–98)	66 (31–98)	57 (29–88)	<0.001
Male	115 (47.7)	61 (53.0)	54 (42.9)	0.11
**Disease severity status**				
General	108 (44.8)	36 (31.3)	72 (57.1)	
Severe	109 (45.2)	60 (52.2)	49 (38.9)	
Critical	24 (10.0)	19 (16.5)	5 (4.0)	<0.001
**Signs and symptoms at admission**				
Time from symptom onset to admission, Median (range), d	55 (5–93)	59 (10–83)	53 (5–93)	0.59
Fever	186 (77.2)	94 (81.7)	92 (73.0)	0.16
Cough	133 (55.2)	62 (53.9)	71 (56.3)	0.70
Shortness of breath	65 (27.0)	38 (33.0)	27 (21.4)	0.04
Fatigue	92 (38.2)	40 (34.8)	52 (41.3)	0.30
Nausea	18 (7.5)	6 (5.2)	12 (9.5)	0.20
Diarrhea	32(13.3)	16 (13.9)	16 (12.7)	0.78
Chest pain	8 (3.6)	2 (1.7)	6 (4.8)	0.19
Sore throat	16 (6.6)	7 (6.1)	9 (7.1)	0.74
Rhinorrhea	8 (3.3)	1 (0.9)	7 (5.6)	0.04
Headache	4 (1.7)	1 (0.9)	3 (2.4)	0.36
**Chronic medical illness**				
Hypertension	98 (40.7)	55 (47.8)	43 (34.1)	0.03
Diabetes	41 (17.0)	22 (19.1)	19 (15.1)	0.40
Cardiovascular disease	39 (16.2)	24 (20.9)	13 (10.3)	0.02
Cerebrovascular disease	13 (5.4)	10 (8.7)	3 (2.4)	0.03
Chronic obstructive pulmonary disease	21 (8.7)	13 (11.3)	8 (6.3)	0.17
Cancer	10 (4.1)	5 (4.3)	5 (4.0)	0.88
Kidney disease	13 (5.4)	9 (2.6)	4 (3.2)	0.11
Hepatic disease	10 (4.1)	3 (2.8)	7 (5.6)	0.25
**Laboratory findings at admission, median (IQR)**				
White blood cell, × 10^9^/L	6.2 (4.7–8.7)	6.4 (4.4–9.5)	5.8 (4.7–7.0)	0.08
Neutrophil, × 10^9^/L	4.1 (2.8–6.7)	5.2 (3.0–8.5)	3.7 (2.6–5.6)	<0.001
Lymphocytes, × 10^9^/L	1.2 (0.8–1.6)	0.9 (0.6–1.2)	1.4 (1.1–1.8)	<0.001
Platelets, × 10^9^/L	213.0 (166.0–267.0)	198.0 (140.0–258.0)	225.0 (181.5–275.0)	0.010
Hemoglobin, g/L	115 (101–129)	110 (95–125)	117 (106–132)	0.006
CRP, mg/L	11.3 (5.0–36.6)	21.4 (5.1–67.5)	5.0 (5.0–24.2)	<0.001
PCT, ng/mL	0.06 (0.03–0.18)	0.12 (0.05–0.63)	0.04 (0.02–0.09)	0.08
CK-MB, ng/mL	1.0 (0.7–2.1)	1.4 (0.8–3.4)	0.9 (0.6–1.5)	0.005
Myoglobin, μg/L	38.5 (25.4–100.9)	49.1 (29.9–171.8)	33.8 (21.7–53.2)	<0.001
hs-TnI, ng/mL	0.006 (<0.006–0.328)	0.027 (<0.006–0.183)	0.006 (<0.0060–0.0065)	0.02
NT-proBNP, pg/L	474 (184–1,840)	1,910 (1,055–5,270)	216.4 (79.1–365.4)	<0.001
ALT, U/L	24.0 (16.0–42.0)	25.5 (17.0–43.8)	22.0 (15.0–40.5)	0.54
AST, U/L	24.0 (17.0–34.0)	25.0 (18.0–40.0)	23.0 (17.0–30.0)	0.18
Albumin, g/dL	37.3 (34.6–40.1)	36.4 (32.8–39.5)	38.9 (36.7–41.2)	0.001
Creatinine, mg/dL	56.0 (48.0–72.0)	56.5 (49.0–82.0)	54.5 (46.0–68.3)	0.004
Potassium, mEq/L	4.3 (3.8–4.6)	4.3 (3.8.4.6)	4.2 (3.9–4.5)	0.27
Sodium, mEq/L	142.0 (139.0.146.0)	141.0 (139.0–145.0)	143 (139.2–146.0)	0.36
d-Dimer, μg/mL	2.4 (0.7–13.7)	6.4 (1.5–20.9)	0.96 (0.43–4.29)	<0.001
**ECG findings**				
ECG abnormalities	65 (27.0)	37 (32.2)	28 (22.2)	0.110
**Treatments**				
Oxygen inhalation	148 (61.4)	89 (77.4)	59 (46.8)	<0.001
Non-invasive ventilation	28 (11.6)	25 (21.7)	3 (2.4)	<0.001
Invasive mechanical ventilation	44 (18.3)	35 (30.4)	9 (7.1)	<0.001
Continuous renal replacement therapy	21 (8.7)	18 (15.7)	3 (2.4)	<0.001
Antiviral treatment	76 (31.5)	44 (38.3)	32 (25.4)	0.032
Glucocorticoids	119 (49.4)	68 (59.1)	51 (40.5)	0.004
Intravenous immunoglobulin therapy	114 (47.3)	63 (54.8)	51 (40.5)	0.026
Antibiotic treatment	70 (29.0)	42 (36.5)	28 (22.2)	0.015

*IQR, interquartile range; CRP, C-reactive protein; CK-MB, creatinine kinase–myocardial band; hs-TnI, high-sensitivity troponin I; NT-proBNP, N-terminal pro–B-type natriuretic peptide; ALT, alanine aminotransferase; AST, aspartate aminotransferase*.

*p < 0.05 was considered statistically significant*.

According to chest CT findings (Table 2), most patients (90.9%) have bilateral pneumonia. There was no difference in GGO between the two groups, but there was more consolidation in the MACE group than in the no-MACE group. Measurement of LAD + LV was significantly larger in patients with MACEs (12.1 ± 1.5 cm vs. 11.4 ± 1.3 cm, *p* < 0.001), comparing with no-MACE group. The CTR >0.5 of the MACE group was more than that of the no-MACE group (58.3% vs. 31.7%, *p* < 0.001). In addition, pleural effusion (41.7% vs. 24.6%, *p* < 0.001), pericardial effusion (39.1% vs. 26.2%, *p* = 0.03), and bronchial wall thickening (11.3% vs. 3.2%, *p* = 0.01) were more likely to occur in MACE group. There was no statistical difference between the two groups in other findings such as fibrosis, reticular pattern, and bronchiectasis (all *p* > 0.05).

**Table 2:** Chest CT findings of 241 patients with COVID-19 on admission.

	**All (*n* = 241)**	**Patients with MACEs (*n* = 115)**	**Patients without MACEs (*n* = 126)**	***p***
Unilateral	22 (9.1)	5 (4.3)	17 (13.5)	
Bilateral	219 (90.9)	110 (95.7)	109 (86.5)	0.01
Ground-glass opacities	219 (90.9)	106 (92.2)	113 (89.7)	0.50
Consolidation	138 (57.3)	78 (67.8)	60 (47.6)	0.002
GGO + consolidation	123 (51.0)	72 (62.6)	51(40.5)	0.001
Lymphadenopathy	42 (17.4)	25 (21.7)	17 (13.5)	0.09
LAD + LV	11.7 ± 1.4	12.1 ± 1.5	11.4 ± 1.3	<0.001
CTR >0.5	107 (44.4)	67 (58.3)	40 (31.7)	<0.001
Pleural effusion	79 (32.8)	48 (41.7)	31 (24.6)	<0.001
Pericardial effusion	78 (32.4)	45 (39.1)	33 (26.2)	0.03
Pleural thickening	98 (40.7)	51 (44.3)	47 (37.3)	0.95
Reticular pattern	42(17.4)	18 (15.7)	24 (19.0)	0.49
Crazy paving	63 (26.1)	38 (33.0)	25 (19.8)	0.02
Air bronchogram	89 (36.9)	55 (47.8)	34 (27.0)	0.001
Bronchial wall thickening	17 (7.1)	13 (11.3)	4 (3.2)	0.01
Bronchiolectasis	74 (30.7)	41 (35.7)	33 (26.2)	0.11
Air bubble sign	43 (17.8)	24 (20.9)	19 (15.1)	0.24
Nodule	11 (4.6)	6 (5.2)	5 (4.0)	0.64
Fibrosis	81 (33.6)	41 (35.7)	40 (31.7)	0.52
Halo sign	8 (3.3)	3 (2.6)	5 (4.0)	0.56

*GGO, ground-glass opacity; CTR, cardiothoracic ratio*.

### Outcome Analysis

**Figure 3:**
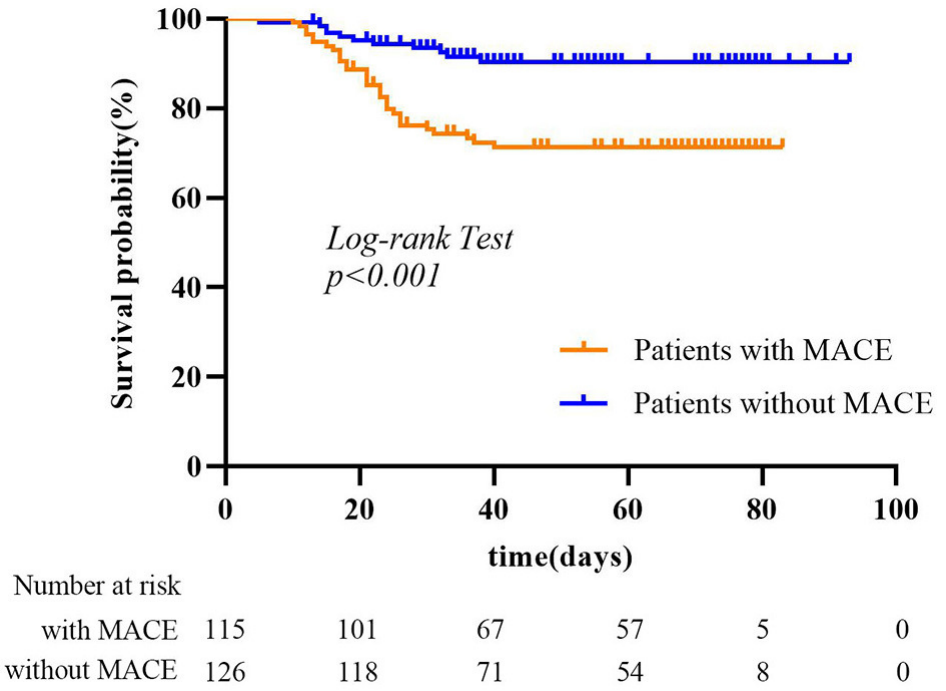
Kaplan–Meier survival curves for patients with MACEs and without MACEs.

The mean follow-up period was 51 days (range, 10–83 days) for the MACE group and 53 days (range, 5–93 days) for no-MACE group. Of the 115 patients, 112 patients (97.4%) had heart failure, 9 patients (7.8%) had acute myocardial infarction, 7 patients (6.1%) had recurrent angina, and 1 patient (0.9%) had ventricular fibrillation. Thirty-two patients (27.8%) died in the MACE group during the follow-up period, much more than the no-MACE group (11 patients, 8.7%). Figure 3 shows Kaplan–Meier survival curves for all-cause mortality in the two groups, which showed patients with MACEs had substantially shorter survival (mortality rate: 27.8% vs. 8.7%, log-rank test: *p* < 0.001). In multivariable analysis, we found that underlying cardiovascular diseases [odds ratio (OR), 28.56; 95% CI, 3.26–250.20; *p* = 0.002) and NT-proBNP (OR, 1.007; 95% CI, 1.004–1.010; *p* < 0.001) were associated with increased odds of MACEs (Table 3). For imaging features, we also found that bronchial wall thickening would increase the odds of MACEs by 13.42 (95% CI, 1.94–93.05; *p* = 0.01). An increase in lymphocyte count and air bronchogram would reduce the odds of MACEs by 0.15 (95% CI, 0.03–0.87; *p* = 0.03) and 0.11 (95% CI, 0.01–0.78; *p* = 0.03), respectively.

**Table 3:** Risk factors associated with MACEs.

**Variables**	**Univariable OR (95% CI)**	***p*-value**	**Multivariable OR (95% CI)**	***p*-value**
**Demographics and clinical characteristics**				
Age	1.05 (1.03–1.07)	<0.001		
Shortness of breath	1.55 (0.31–1.98)	0.04		
Hypertension	1.77 (1.05– 2.97)	0.03		
Diabetes	0.75 (0.38–1.47)	0.40		
Cardiovascular disease	2.29 (1.11–4.75)	0.03	28.56 (3.26–250.20)	0.002
Cerebrovascular disease	3.91 (1.05–14.56)	0.04		
**Laboratory findings at admission**				
Neutrophil, × 10^9^**/**L	1.14 (1.06–1.24)	0.001		
Lymphocytes, × 10^9^**/**L	0.18 (0.10–0.32)	<0.001	0.15 (0.03–0.87)	0.03
Platelets, × 10^9^**/**L	0.996 (0.993–0.999)	0.01		
Hemoglobin, g/L	0.983 (0.970–0.995)	0.01		
CRP, mg/L	1.01 (1.01–1.02)	0.001		
CK-MB, ng/mL	1.16 (1.05–1.29)	0.004		
Myoglobin, μg/L	1.003 (1.001–1.005)	<0.001		
hs-TnI, pg/L	4.25 (1.61–9.46)	<0.001		
NT-proBNP, pg/L	1.007 (1.005–1.010)	<0.001	1.007 (1.004–1.010)	<0.001
Albumin, g/dL	0.91 (0.86–0.96)	0.002		
Creatinine, mg/dL	1.01 (1.00–1.02)	0.01		
d-Dimer, μg/mL	1.03 (1.01–1.04)	0.002		
**Imaging features**				
Bilateral	3.43 (1.22–9.63)	0.02		
Consolidation	2.32 (1.37–3.92)	0.002		
GGO + consolidation	2.46 (1.47–4.14)	0.001		
Pleural effusion	6.37 (3.60–11.27)	<0.001		
Pericardial effusion	2.10 (1.26–3.53)	0.01		
Crazy paving	1.99 (1.11–3.58)	0.02		
Air bronchogram	2.45 (1.43–4.20)	0.001	0.11 (0.01–0.78)	0.03
Bronchial wall thickening	3.89 (1.23–12.29)	0.02	13.42 (1.94–93.05)	0.01
LAD + LV	1.49 (1.22–1.83)	<0.001		
CTR >0.5	2.35 (1.39–3.96)	0.001		

*CRP, C-reactive protein; CK-MB, creatinine kinase–myocardial band; hs-TnI, high-sensitivity troponin I; NT-proBNP, N-terminal pro–B-type natriuretic peptide; GGO, ground-glass opacity; CTR, cardiothoracic ratio*.

### Prediction for MACEs

**Figure 4:**
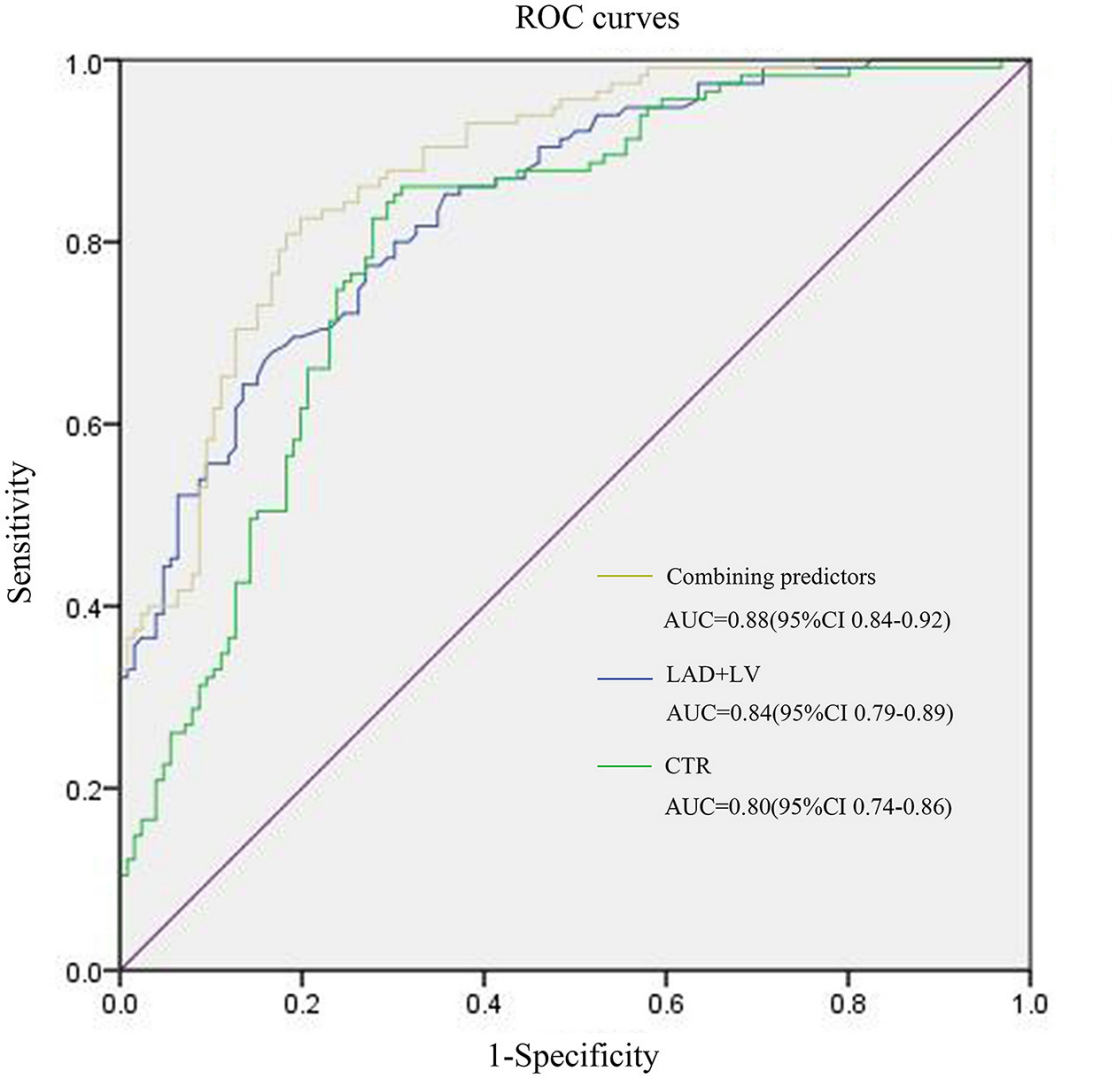
ROC curves for MACE prediction of patients with COVID-19. LAD, The anteroposterior diameter of the left atrium; LV, The length of the left ventricle; CTR, cardiothoracic ratio.

**Figure 5:**
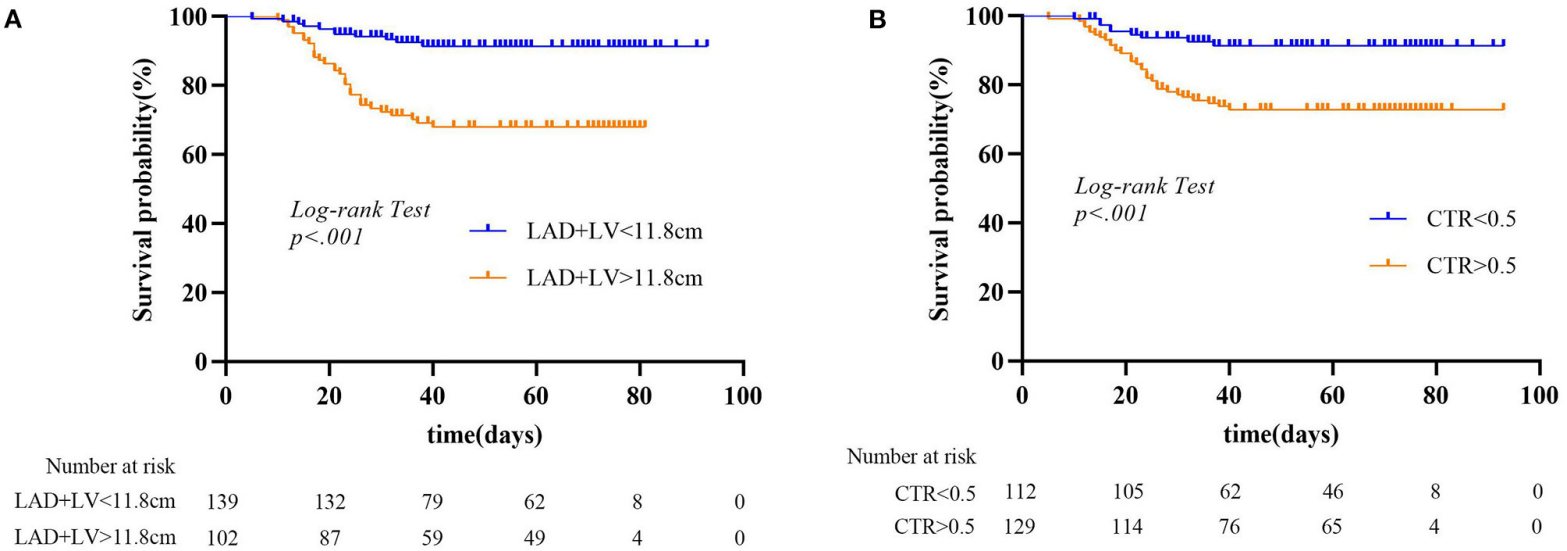
**(A,B)** Kaplan–Meier curves for chest CT parameters and all-cause mortality endpoint. LAD, anteroposterior diameter of the left atrium; LV, length of the left ventricle; CTR, cardiothoracic ratio.

For MACE prediction analysis (Figure 4), the areas under the curve (AUCs) of ROC were 0.84 (*p* < 0.001) in LAD + LV and 0.8 (*p* < 0.001) in CTR. The cutoff value of LAD + LV was 11.8 cm by ROC curve, yielding a sensitivity of 67.8%, a specificity of 83.3%, a positive predictive value of 60%, and a negative predictive value of 73.8%. CTR >0.5 yielded a sensitivity of 86.1% and a specificity of 69.0%. Our results showed that combining predictors (LAD + LV and CTR) was the best predictor for MACEs (AUC = 0.88, *p* < 0.001), which was a little higher than a single variable. The sensitivity and specificity were 82.6% and 80.2%, with a cutoff value of 12.1 cm and 0.51. Using LAD + LV (Figure 5A) and CTR > 0.5 (Figure 5B) was also helpful for classifying subjects at high risk of death. Mortality rates were significantly higher in patients with LAD + LV >11.8 cm and CTR >0.5 than in patients with LAD + LV <11.8 cm and CTR <0.5 (log-rank test: *p* < 0.001).

**Figure 6:**
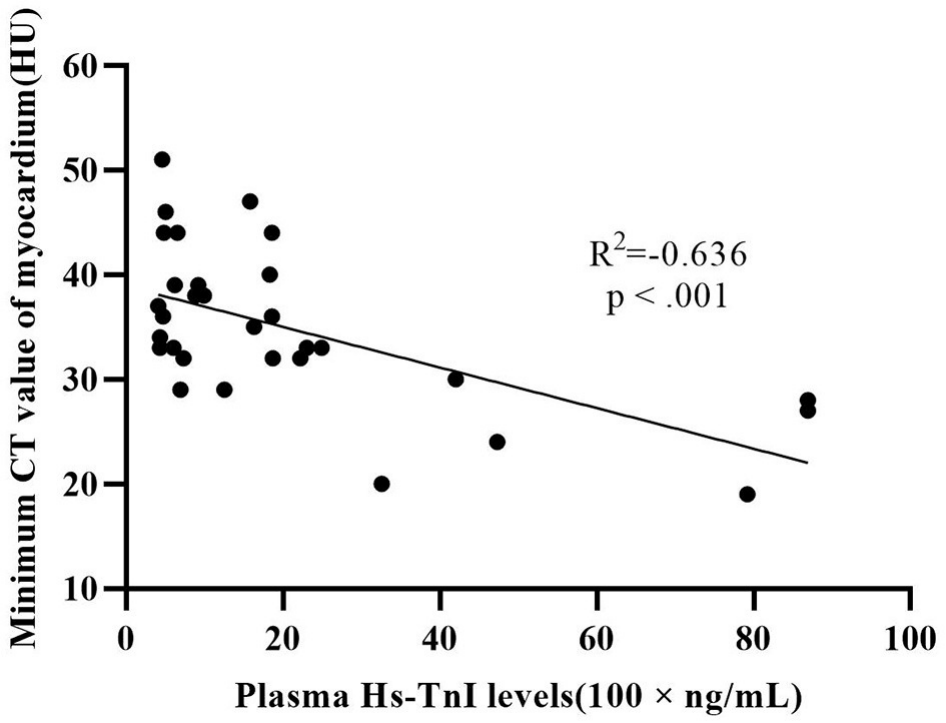
Correlation between plasma hs-TnI and minimum CT value of myocardium.

There were 32 patients with cardiac injury in our study population. The difference of minimum CT value (34.5 ± 8.3 HU vs. 44.5 ± 6.3 HU, *p* < 0.001) and mean CT value (42.5 ± 7.1 HU vs. 47.9 ± 6.0 HU, *p* ≤ 0.008) between patients with cardiac injury and patients without cardiac injury was statistically significant. The lateral wall of LV was more vulnerable compared to septum and anterior wall in our study. Plasma hs-TnI levels in patients with cardiac injury showed a moderate negative correlation with minimum CT value (*R*^2^ = −0.636, *p* < 0.001) (Figure 6).

## Discussion

The present study demonstrates the influence of adverse cardiovascular events on the prognosis of patients with COVID-19. During hospitalization, patients were more likely to have MACEs (47.7%), the highest of which was heart failure, followed by ischemic events and malignant arrhythmias. Chest CT plain scan is an effective method to screen the high-risk population of cardiovascular adverse events.

Cardiac-related complications are common in patients with pneumonia. Approximately 3% of inpatients with pneumonia may have cardiac arrest (13). In our research, patients with MACEs are older and more likely to experience shortness of breath when admitted. In addition to abnormal cardiac markers, patients with MACEs also have anemia, decreased albumin, and coagulation disorders. In terms of treatment, patients with MACEs need more therapy including respiratory support, intravenous immunoglobulin therapy, glucocorticoids, and so on. Similar results were obtained in another study by Shi et al. (6). This observation suggests that cardiac-related injury is possibly associated with the clinical outcomes of COVID-19. A recent study shows that patients who received care in the intensive care unit (ICU) were more likely to have cardiac injury (22.2%) than non-ICU patients (14).

In terms of imaging features, there was no statistical difference in GGO between the two groups, but the MACE group had more consolidation. Consolidation is usually presented in 2–64% COVID-19 patients (15, 16). It may relate to cellular fibromyxoid exudates in alveoli and was considered as an indication of disease progression. The incidence rates of pleural effusion, pericardial effusion, and bronchial wall thickening were higher in the MACE group, which may indicate the occurrence of severe inflammation. A recent study showed that such signs were more likely to occur in severe patients (17).

In this retrospective study, we analyzed the clinical and imaging characteristics of patients with MACEs and put forward an optimal risk prediction model to explore the risk factors. We found patients with cardiovascular diseases, low lymphocytes count, high NT-proBNP, and bronchial wall thickening were independent risk factors for MACEs. It is reasonable to speculate that patients with underlying cardiovascular diseases are more likely to suffer from cardiac injury, which eventually leads to deterioration. Based on the experience of influenza (18) and other acute inflammatory conditions (19), patients with long-term coronary heart disease and those with risk factors for atherosclerotic cardiovascular disease have a higher risk of acute coronary syndrome during acute infection. In systemic inflammatory response, the inflammatory activity in coronary atherosclerotic plaques is intensified and easy to rupture (20). Meanwhile, endothelial dysfunction and blood procoagulant activity are increased to form a thrombus (21). Shi et al. found that patients with cardiac injury have a high level of the inflammatory response, such as C-reactive protein, procalcitonin, and leukocytes. Our results were similar, but there was no significant difference in PCT between the two groups.

In this study, heart failure accounted for a large proportion of adverse cardiovascular events. The clinical classification of cardiac function is of great value for guiding treatment and evaluating prognosis. Cardiac function mainly depends on laboratory results and clinical manifestations, and there is little research to screen heart failure by chest CT. LAD + LV is used as an index to evaluate the size of the heart, which is a direct sign of heart failure. As novel coronavirus pneumonia was spread rapidly, lack of medical resources is a problem that every country has to face. Almost all patients will do a general chest CT plain scan to understand the severity of the disease to guide the treatment. Some laboratory tests of heart injury such as hs-TnI are usually performed only when the patient has symptoms or abnormal electrocardiogram (ECG). If a part of patients can be screened by CT plain scan, it will greatly improve the efficiency of clinical diagnosis and save resources. Our study showed that the sensitivity, specificity, and positive and negative predictive values of LAD + LV were 67.8%, 83.3%, and 60% and 73.8%, respectively. At present, the CTR is measured by X-ray routinely at home and abroad, although it is simple and practical, geometric magnification and distortion can be usually seen. We also have good results in screening MACE patients that CTR >0.5 yielded a sensitivity of 86.1% and a specificity of 69%. This method is also very friendly for patients with poor renal function, as they cannot have an enhancement CT scan. To some extent, they can also reflect the prognosis of MACE patients.

In the current study, plasma hs-TnI levels were significantly negatively linearly correlated with minimum CT values of the myocardium, indicating that measurement of myocardial CT value can reflect the degree of myocardial injury to a certain extent. The mechanisms leading to cardiac damage are numerous and include direct insult to myocytes by the virus, cytokine and interferon inflammatory responses, myocardial interstitial fibrotic response, and T1 and T2 helper cell response. Inflammation leads to increased permeability of cell membrane and cell edema. CT is sensitive to edema, so we can screen patients with myocardial damage through plain CT. This may provide a new method for early screening of patients with myocardial injury.

The present study provided a new way to screen patients with myocardial injury using chest plain CT. Non-contrast chest CT is an examination performed by every COVID-19 patient in order to observe the lesions of the lung. By measuring the CTR, LAD + LV, and CT value of COVID-19 patients with chest plain CT, the heart of patients can be preliminarily evaluated, and the patients who may have heart injury can be screened for further examination such as echocardiography or cardiac magnetic resonance. Cardiac magnetic resonance can evaluate not only the structure and function of the heart, but also the histological characteristics of the myocardium. Late enhancement, T1/T2 mapping, and strain can evaluate myocardial injury in detail, such as edema, fibrosis, and myocardial movement. This method not only contributes to early diagnosis and treatment, but also saves medical resources in the current medical environment.

Our study has some limitations. First, this is a single-center study with a short follow-up time. Larger sample size and longer follow-up time would be the direction of future research to validate the results of our study. Second, the measurement of LAD + LV has the following defects: neglecting the difference of wall thickness and time phase of the heart and neglecting the difference of gender and body mass index. The patients were enrolled when the disease spread fast, and the number of patients grew rapidly; thus, some demographic information such as height and weight was missing. So, we cannot calculate the index value. However, after adjusting for age and gender using analysis of covariance, there were still significant differences in LA + LV between two groups (*p* = 0.042). But we considered it as a preliminary screening of patients with MACEs, which we think is appropriate. Third, the measurement of myocardial CT value is affected by many factors, such as artifacts. Fourth, we did not have the data of echocardiography or New York Heart Association (NYHA) classification. Only patients with myocardial injury will be examined by echocardiography to obtain ejection fraction value, and physicians will evaluate NYHA classification. Therefore, not all patients in our group have these data. In addition, right heart, which could also be affected in COVID-19 patients, was not analyzed in our study.

### Conclusion

Cardiac injury is a common condition among patients hospitalized with COVID-19, and it is associated with a higher risk of in-hospital mortality. Chest CT plain scan can effectively screen a part of high-risk patients, so as to guide doctors to treat early and improve prognosis. This method can also save medical resources better in the current environment.

## Data Availability Statement

The raw data supporting the conclusions of this article will be made available by the authors, without undue reservation.

## Ethics Statement

The studies involving human participants were reviewed and approved by Research Ethics Committee of the Renmin Hospital of Wuhan University, Wuhan, China (approval number: WDRY2020-K038). The Ethics Committee waived the requirement of written informed consent for participation.

## Author Contributions

SL: wrote the paper. XW, HH, BH, HY, and QZ: collect the patient. JX, JH, and WY: collect the data. YL, HZha, and TL: search the reference. KH, YZ, and ZH: do the statistics. HZhu and BZ: revise the manuscript. SZ, AS, AA, JC, XZ, and ML: guide the research. All authors contributed to the article and approved the submitted version.

## Funding

ML was funded by Construction Research Project of Key Laboratory (Cultivation) of Chinese Academy of Medical Sciences (2019PT310025), National Natural Science Foundation of China (Grant Nos. 81971588, 81771811, and 81970331), Capital Clinically Characteristic Applied Research Fund (Grant No. Z191100006619021), and Clinical and Translational Fund of Chinese Academy of Medical Sciences (2019XK320063). XZ was funded by National Natural Science Foundation of China (Grant No. 81970331).

## Conflict of Interest

The authors declare that the research was conducted in the absence of any commercial or financial relationships that could be construed as a potential conflict of interest.

## Publisher's Note

All claims expressed in this article are solely those of the authors and do not necessarily represent those of their affiliated organizations, or those of the publisher, the editors and the reviewers. Any product that may be evaluated in this article, or claim that may be made by its manufacturer, is not guaranteed or endorsed by the publisher.

## Abbreviations

COVID-19: coronavirus disease 2019
MACE: major adverse cardiovascular events
LV: left ventricle
CTR: cardiothoracic ratio
GGO: ground-glass opacity
LAD: diameter of the left atrium
hs-TnI: high-sensitivity troponin I
NT-proBNP: N-terminal pro–B-type natriuretic peptide.
